# Supplementary material for: Causal Relationship between Aging and Anorexia Nervosa: A White-Matter-Microstructure-Mediated Mendelian Randomization Analysis
Source: Biomedicines. 2024 Aug 16;12(8):1874. doi: 10.3390/biomedicines12081874 (PMC11351342; doi:10.3390/biomedicines12081874)
Supplement: Supplementary file 1 [file biomedicines-12-01874-s001.zip › Supplementary Figure/Figure S4/Singe SNP plot of MR analysis for TL to ubm-b-2067.pdf]

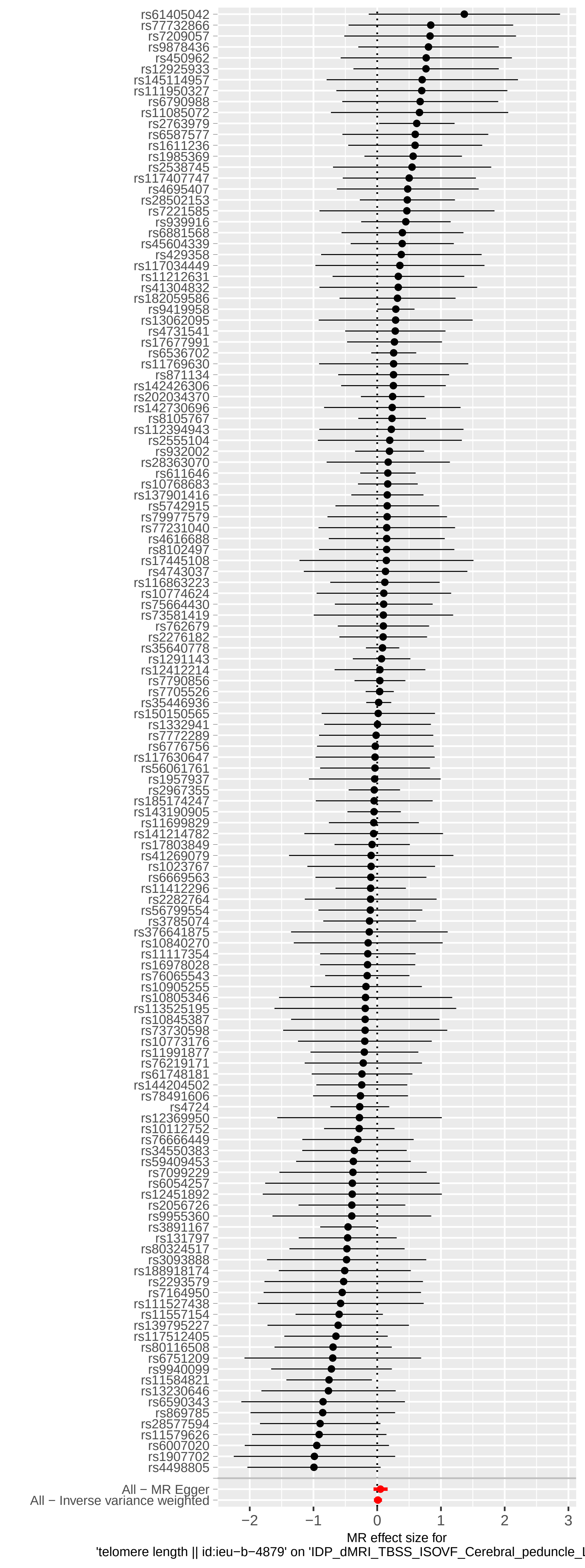
rs61405042
rs77732866
rs7209057
rs9878436
rs450962
rs12925933
rs145114957
rs111950327
rs6790988
rs11085072
rs2763979
rs6587577
rs1611236
rs1985369
rs2538745
rs117407747
rs4695407
rs28502153
rs7221585
rs939916
rs6881568
rs45604339
rs429358
rs117034449
rs11212631
rs41304832
rs182059586
rs9419958
rs13062095
rs4731541
rs17677991
rs6536702
rs11769630
rs871134
rs142426306
rs202034370
rs142730696
rs8105767
rs112394943
rs2555104
rs932002
rs28363070
rs611646
rs10768683
rs137901416
rs5742915
rs79977579
rs77231040
rs4616688
rs8102497
rs17445108
rs4743037
rs116863223
rs10774624
rs75664430
rs73581419
rs762679
rs2276182
rs35640778
rs1291143
rs12412214
rs7790856
rs7705526
rs35446936
rs150150565
rs1332941
rs7772289
rs6776756
rs117630647
rs56061761
rs1957937
rs2967355
rs185174247
rs143190905
rs11699829
rs141214782
rs17803849
rs41269079
rs1023767
rs6669563
rs11412296
rs2282764
rs56799554
rs3785074
rs376641875
rs10840270
rs11117354
rs16978028
rs76065543
rs10905255
rs10805346
rs113525195
rs10845387
rs73730598
rs10773176
rs11991877
rs76219171
rs61748181
rs144204502
rs78491606
rs4724
rs12369950
rs10112752
rs76666449
rs34550383
rs59409453
rs7099229
rs6054257
rs12451892
rs2056726
rs9955360
rs3891167
rs131797
rs80324517
rs3093888
rs188918174
rs2293579
rs7164950
rs111527438
rs11557154
rs139795227
rs117512405
rs80116508
rs6751209
rs9940099
rs11584821
rs13230646
rs6590343
rs869785
rs28577594
rs11579626
rs6007020
rs1907702
rs4498805
All − MR Egger
All − Inverse variance weighted
−2
−1
0
1
2
3
MR effect size for
'telomere length || id:ieu−b−4879' on 'IDP_dMRI_TBSS_ISOVF_Cerebral_peduncle_l
